# Supplementary material for: Delay in Flowering Time in Arabidopsis thaliana Col-0 Under Water Deficit and in the ddc Triple Methylation Knockout Mutant Is Correlated with Shared Overexpression of BBX16 and BBX17
Source: Int J Mol Sci. 2025 Aug 28;26(17):8360. doi: 10.3390/ijms26178360 (PMC12428429; doi:10.3390/ijms26178360)
Supplement: Supplementary file 1 [file ijms-26-08360-s001.zip › Supplementary Figures.pdf]

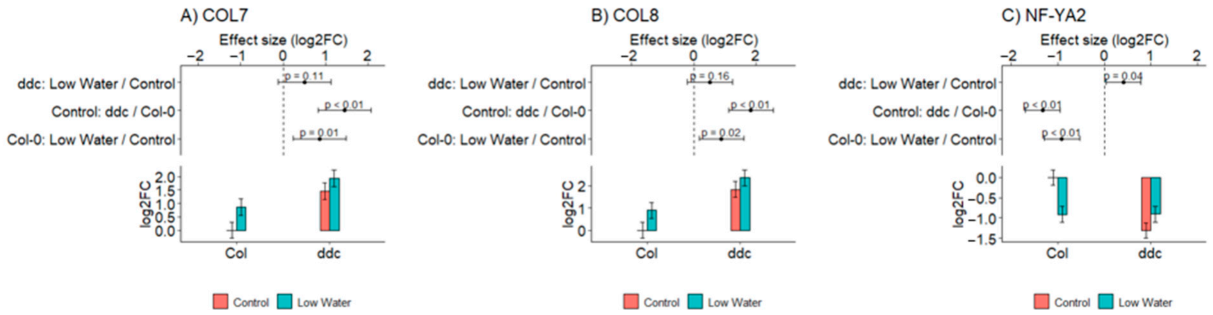

Supplementary Figure S1 Differential expression of A) *COL7/BBX16;* B) *COL8/BBX17*; and C) *NF-YA2* confirmed by qRT-PCR, together with effect sizes of the difference between three pairwise comparisons, confirmed by a linear regression model.

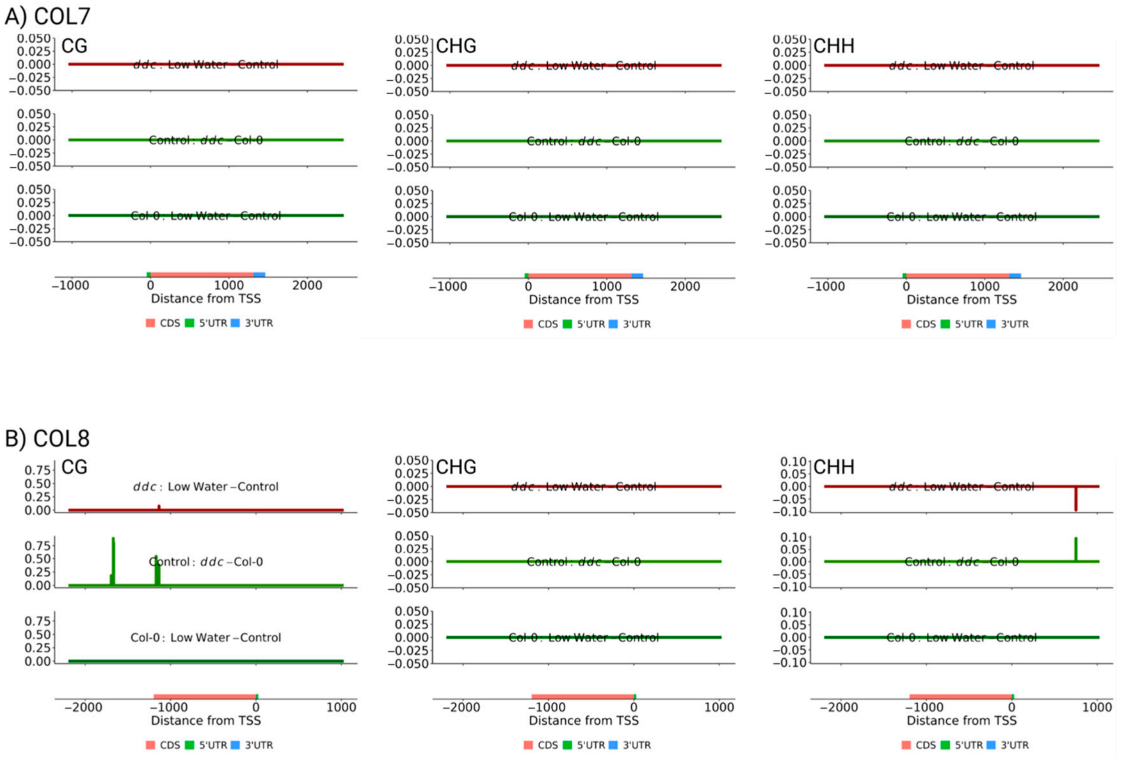

Supplementary Figure S2 Differentially methylated loci (DML) of CG (left), CHG (middle) and CHH (right) contexts, calculated with linear mixed model from three biological replicates for A) COL7 and B) COL8. On the x axis, transcription start site is labeled as 0 with position numbers increasing according to the positive DNA strand. Y axis measures differences in cytosine methylation proportion for each individual cytosine for the respective comparison. The three pairwise comparisons are presented from top to bottom as follows: *ddc*: Low Water – Control; Control: *ddc* – Col-0; Col-0: Low Water – Control.

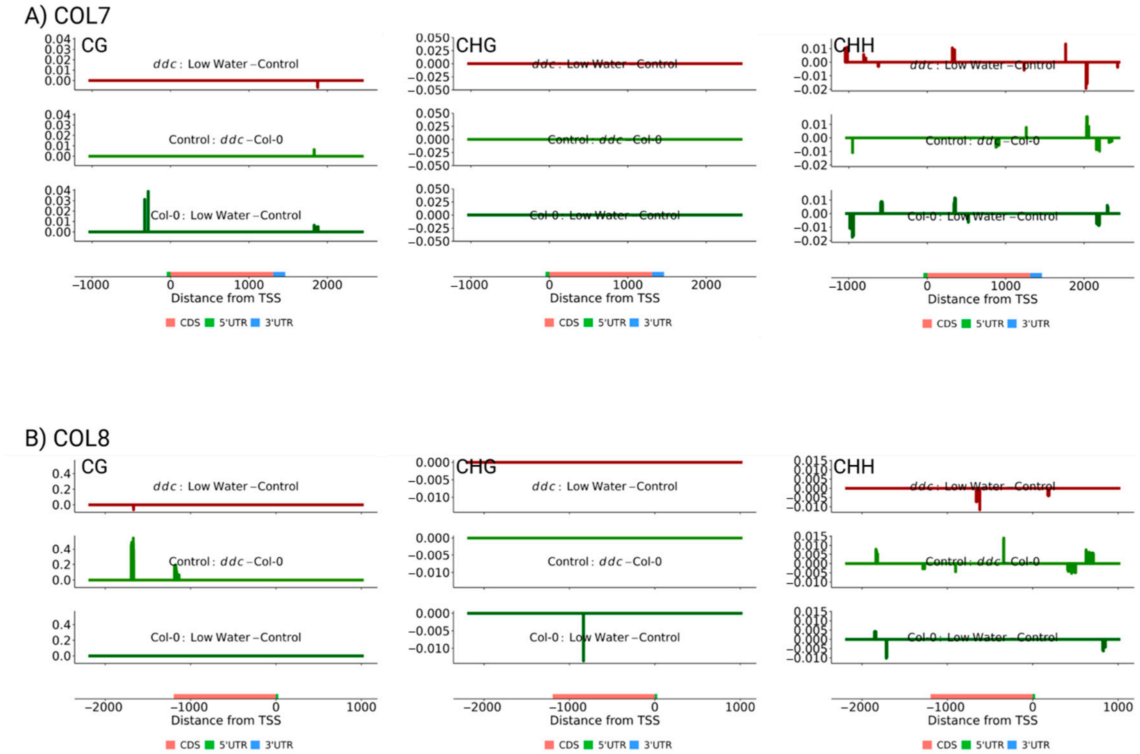

Supplementary Figure S3 Differentially methylated regions (DMR) of CG (left), CHG (middle) and CHH (right) contexts, calculated with linear mixed model from three biological replicates for A) *COL7* and B) *COL8*. On the x axis, transcription start site is labeled as 0 with position numbers increasing according to the positive DNA strand. Y axis measures differences in cytosine methylation proportion for each individual cytosine for the respective comparison. The three pairwise comparisons are presented from top to bottom as follows: *ddc*: Low Water – Control; Control: *ddc* – Col-0; Col-0: Low Water – Control.

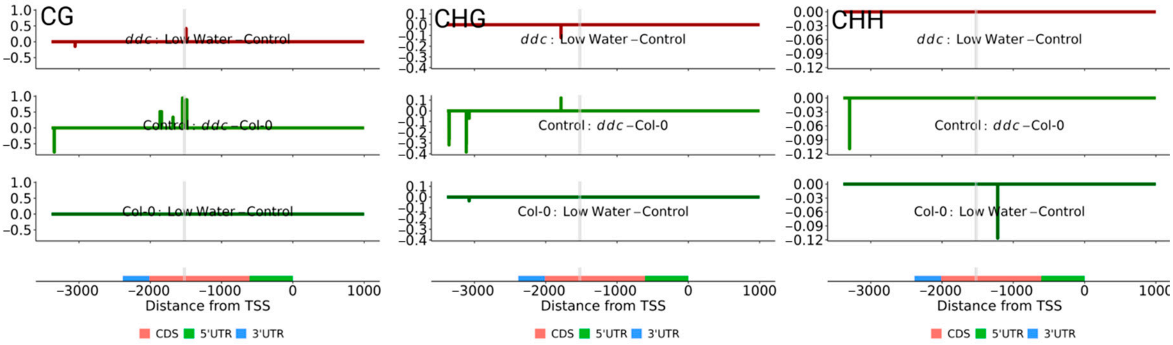

Supplementary Figure S4 Differentially methylated loci (DML) of CG (left), CHG (middle) and CHH (right) contexts, calculated with linear mixed model from three biological replicates for *NF-YA2*. On the x axis, transcription start site is labeled as 0 with position numbers increasing according to the positive DNA strand. Y axis measures differences in cytosine methylation proportion for each individual cytosine for the respective comparison. The three pairwise comparisons are presented from top to bottom as follows: *ddc*: Low Water – Control; Control: *ddc* – Col-0; Col-0: Low Water – Control. Light grey lines indicate the target site of *miR169d*.

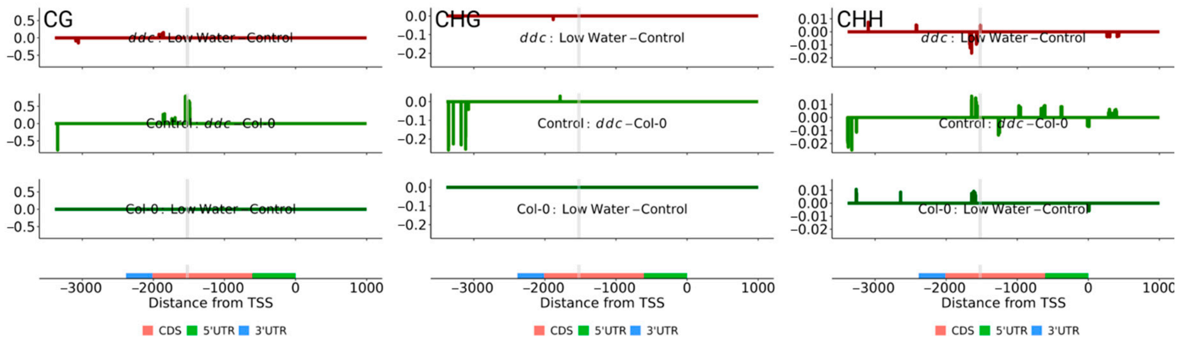

Supplementary Figure S5 Differentially methylated regions (DMR) of CG (left), CHG (middle) and CHH (right) contexts, calculated with linear mixed model from three biological replicates for *NF-YA2*. On the x axis, transcription start site is labeled as 0 with position numbers increasing according to the positive DNA strand. Y axis measures differences in cytosine methylation proportion for each individual cytosine for the respective comparison. The three pairwise comparisons are presented from top to bottom as follows: *ddc*: Low Water – Control; Control: *ddc* – Col-0; Col-0: Low Water – Control. Light grey lines indicate the target site of *miR169d*.
